# Endogenous tRNA‐derived small RNA (tRF3‐Thr‐AGT) inhibits ZBP1/NLRP3 pathway‐mediated cell pyroptosis to attenuate acute pancreatitis (AP)

**DOI:** 10.1111/jcmm.16972

**Published:** 2021-10-13

**Authors:** Boshi Sun, Zhuomiaoyu Chen, Qiang Chi, Yifan Zhang, Bo Gao

**Affiliations:** ^1^ The 3rd Department of General Surgery The 2ndAffiliated Hospital of Harbin Medical University Harbin, Heilongjiang China; ^2^ Department of General Surgery Peking University People’s Hospital Beijing China

**Keywords:** acute pancreatitis, cell pyroptosis, transfer RNA‐derived small RNAs, tRF3‐Thr‐AGT, Z‐DNA‐binding protein 1

## Abstract

Endogenous transfer RNA‐derived small RNAs (tsRNAs) are newly identified RNAs that are closely associated with the pathogenesis of multiple diseases, but the involvement of tsRNAs in regulating acute pancreatitis (AP) development has not been reported. In this study, we screened out a novel tsRNA, tRF3‐Thr‐AGT, that was aberrantly downregulated in the acinar cell line AR42J treated with sodium taurocholate (STC) and the pancreatic tissues of STC‐induced AP rat models. In addition, STC treatment suppressed cell viability, induced pyroptotic cell death and cellular inflammation in AP models in vitro and in vivo. Overexpression of tRF3‐Thr‐AGT partially reversed STC‐induced detrimental effects on the AR42J cells. Next, Z‐DNA‐binding protein 1 (ZBP1) was identified as the downstream target of tRF3‐Thr‐AGT. Interestingly, upregulation of tRF3‐Thr‐AGT suppressed NOD‐like receptor protein 3 (NLRP3)‐mediated pyroptotic cell death in STC‐treated AR42J cells via degrading ZBP1. Moreover, the effects of tRF3‐Thr‐AGT overexpression on cell viability and inflammation in AR42J cells were abrogated by upregulating ZBP1 and NLRP3. Collectively, our data indicated that tRF3‐Thr‐AGT suppressed ZBP1 expressions to restrain NLRP3‐mediated pyroptotic cell death and inflammation in AP models. This study, for the first time, identified the role and potential underlying mechanisms by which tRF3‐Thr‐AGT regulated AP pathogenesis.

## 1 BACKGROUND

Acute pancreatitis (AP) is a common inflammatory disease occurring in pancreas, and 80% of AP patients are diagnosed as mild AP (MAP), which is characterized with self‐limitation and better prognosis, whereas about 20% of AP patients are determined as severe AP (SAP) with high mortality.^1^, ^2^, ^3^ As indicated in the previous studies, the pathogenesis of AP is complicated and it is reported that abnormal activation of pancreatic enzymes^4^, ^5^ and super‐inflammation^6^, ^7^ are two pathogenic factors that contribute to AP progression, but the unknown underlying mechanisms seriously limit the development of effective treatment strategies for AP in clinic.^8^ According to the previous publications, various types of cell death, including cell apoptosis,^9^, ^10^ necroptosis,^1^, ^11^ ferroptosis^12^, ^13^ and pyroptosis,^14^, ^15^ facilitate the development of AP. Amongst them, cell pyroptosis is a type of inflammation‐associated cell death,^16^, ^17^ which is characterized with acute inflammatory reactions and is reported to play critical role to aggravate AP.^14^, ^15^ For example, according to the data from Wang et al.,^14^ Gasdermin D (GSDMD)‐mediated pyroptotic cell death and inflammatory cytokines secretion contribute to SAP pathogenesis, and Lin et al. evidence that blockage of cell pyroptosis is effective to ameliorate AP.^15^

Recently, a large variety of AP‐associated non‐coding RNAs (ncRNAs) with post‐transcriptional regulation activities are identified, those ncRNAs include long non‐coding RNAs (LncRNAs),^18^, ^19^ circular RNAs (circRNAs)^20^, ^21^ and microRNAs (miRNAs).^22^, ^23^ In addition to the above ncRNAs, a newly identified endogenous transfer RNA (tRNA)‐derived small RNAs (tsRNAs) are reported to be associated with multiple diseases, such as Alzheimer's disease,^24^ IgA nephropathy^25^ and tumours.^26^ However, the role of tsRNAs in regulating AP pathogenesis has not been investigated. As previously reported, the main functions of tRNAs are to transfer the amino acid for gene translation,^27^, ^28^ and aside from that, researchers report that tRNAs are fragmented into RNA fragments under some specific conditions. Those RNA fragments are named as tRNA‐derived RNA fragments (tRFs) and half‐tRNAs (tiRNAs), which are able to regulate gene expressions.^26^, ^29^, ^30^ In our preliminary work, we screened out a novel tsRNA tRF3‐Thr‐AGT that was closely associated with AP, indicating that this tsRNA might be crucial for AP development. Thus, this study selected tRF3‐Thr‐AGT for further investigations.

Recent data suggest that tsRNAs regulate cellular functions by targeting the 3′ untranslated region (3′UTR) of their downstream target genes, which is similar to miRNAs.^31^ Interestingly, data from Zhang et al. hint that tsRNAs act as competing endogenous RNA (ceRNA) to sponge miRNAs.^32^ By performing the bioinformatics analysis, we predicted that tRF3‐Thr‐AGT was capable of binding to the 3′UTR of Z‐DNA‐binding protein 1 (ZBP1), indicating that ZBP1 could be potentially targeted by tRF3‐Thr‐AGT. In addition, ZBP1 is closely associated with inflammation^33^ and cell pyroptosis.^34^, ^35^ For example, Szczesny et al. evidence that ZBP1 promotes oxidative stress‐mediated inflammation in epithelial cells,^33^ and data from other teams show that ZBP1 activates NLRP3 inflammasome‐mediated pyroptotic cell death.^34^, ^35^ Taken together the above information, this study was designed to investigate the role and potential underlying mechanisms of a novel tsRNA tRF3‐Thr‐AGT in regulating AP pathogenesis.

## 2 MATERIALS AND METHODS

### 2.1 Cell culture, vectors transfection and treatments

The rat pancreatic acinar AR42J cells were obtained from American Type Culture Collection (ATCC, USA) and the cells were cultured in the Ham's F‐12K medium (Gibco, USA) supplemented with 10% foetal bovine serum (FBS, Gibco, USA) as previously reported.^36^ The tRF3‐Thr‐AGT mimic and inhibitor, and overexpression vectors for ZBP1 and NLRP3 were designed and constructed by a commercial third‐party company (Sangon Biotech, Shanghai, China), and the above vectors were delivered into the AR42J cells by using the Lipofectamine 3000 Vector Transfection reagent purchased from QIAGEN (CA, USA) in keeping with the producer's protocol. The sequences for tRF3‐Thr‐AGT mimic and inhibitor were shown as follows: tRF3‐Thr‐AGT mimic (5′‐AUC CCA GCG GUG CCU CC‐3′), tRF3‐Thr‐AGT inhibitor (5′‐GGA GGC ACC GCU GGG AU‐3′). Moreover, the lentivirus expressing rat ZBP1 and NLRP3 were synthesized by Sangon Biotech (Shanghai, China). The small interfering RNA (siRNA) for NLRP3 (5′‐GCA GGU UCU ACU CUA UCA AGG‐3′) was transfected into cells for its knockdown. To establish the cellular AP models, the AR42J cells were subjected to 200 μM/L STC (Sigma, MO, USA) exposure for 0, 6, 12, 24 and 48 h, respectively.

### 2.2 Real‐time qPCR

Total RNA was extracted from AR42J cells and rat pancreatic tissues by using the TRIzol reagent (Invitrogen, CA, USA), and the quality of the RNA was guaranteed by performing the agarose electrophoresis. To quantify tRF3‐Thr‐AGT levels, the rtStar^TM^ tRF and tiRNA pretreatment kit (Arraystar, USA) was initially used to remove excess modifications, and the rtStar^TM^ First‐strand cDNA Synthesis kit (Arraystar, USA) was used to reversely transcribe RNA into complementary DNA (cDNA) in keeping with the manufacturer's protocol. For other genes, the cDNA was obtained through using a reverse transcription kit purchased from Invitrogen (CA, USA). Then, an SYBR Green PCR kit (Takara, Japan) was employed to quantify the expression levels of the genes at mRNA levels, tRF3‐Thr‐AGT was normalized by U6, and other genes were normalized by GAPDH. The primer sequences for the target genes, including tRF3‐Thr‐AGT, U6, IL‐6, IL‐13, TNF‐α, IL‐1β, IL‐18 and GAPDH, are, respectively, constructed and listed in Table 1. The primers for tRF3‐Thr‐AGT were designed according to the previous publications.^37^, ^38^, ^39^ In brief, the forward primer was designed by using the tRF3‐Thr‐AGT sequence itself. Of note, the Poly (A) tail was added to the tRF3‐Thr‐AGT for reverse transcription, thus, the reverse primer (5′‐GGCCAACCGCGAGAAGATG‐3′) was designed according to the sequence in the Poly (A) tail.

**TABLE 1:** The primer sequences for Real‐Time qPCR

	Primer sequences
tRF3‐Thr‐AGT	Forward: 5′‐ ATCCCAGCGGTGCCTCC‐3′
	Reverse: 5′‐GGCCAACCGCGAGAAGATG‐3′
U6	Forward: 5′‐GCTTCGGCAGCACATATACTA‐3′
	Reverse: 5′‐CGCTTCAGAATTTGCGTGTCAT‐3′
IL‐6	Forward: 5′‐CAAATTCGGTACATCCTC‐3′
	Reverse: 5′‐CTGGCTTGTTCCTCACTA‐3′
IL‐13	Forward: 5′‐GCCAGCCCACAGTTCTAC‐3′
	Reverse: 5′‐GAGATGTTGGTCAGGGAAT‐3′
TNF‐α	Forward: 5′‐CACGCTCTTCTGCCTGCT‐3′
	Reverse: 5′‐GCTTGTCACTCGGGGTTC‐3′
IL‐1β	Forward: 5′‐GGCAGGTGGTATCGATCATC‐3′
	Reverse: 5′‐CACCTTGGATTTGACTTCTA‐3′
IL‐18	Forward: 5′‐GCTGGCTGTAACCCTCTCTG‐3′
	Reverse: 5′‐TTCCTCCTTTTGGCAAGCTA‐3′
GAPDH	Forward: 5′‐TGTTGACATCAATGACCCCTT‐3′
	Reverse: 5′‐CTCCACGACGTACTCAGCG‐3′

### 2.3 Western Blot analysis

The total proteins in the cells and rat tissues were extracted by using the RIPA lysis buffer (Beyotime Biotechnology, Shanghai, China), and the BCA assay kit (Thermo Fisher Scientific, USA) was used to measure protein concentrations. The target proteins in the lysates were separated by using 10% SDS‐PAGE according to their molecular weight, and the proteins were transferred onto PVDF membranes (Millipore, USA), which were further blocked with 5% non‐fat milk. The membranes were then incubated with the primary antibodies against NLRP3 (1:1500, Cell Signaling Technology, MA, USA), ASC (1:2000, Cell Signaling Technology, MA, USA), Gasdermin D (1:2000, Cell Signaling Technology, MA, USA), ZBP1 (1:1500, Cell Signaling Technology, MA, USA) and GAPDH (1:1500, Cell Signaling Technology, MA, USA) at 4℃ overnight and were subsequently probed with the secondary antibody.

### 2.4 RNA sequencing for tsRNAs

The tsRNA sequencing experiments were conducted by Aksomics (Shanghai, China). Briefly, the AR42J cells were treated with 200 μM/L of STC for 30 min, and the live cells were selected for further analysis. The total RNA (2 μg) extracted from the STC‐treated AR42J cells was prepared, and the agarose gel electrophoresis and Nanodrop™ instrument were used to check the integrity and quantity of those RNA samples. Before library preparation, the total RNA samples were treated with 3′‐aminoacyl (charged) deacylation to 3′‐OH for 3′ adaptor ligation, 3′‐cP (2′,3′‐cyclic phosphate) removal to 3′‐OH for 3′ adaptor ligation, 5′‐OH (hydroxyl group) phosphorylation to 5′‐P for 5′‐adaptor ligation, m1A and m3C demethylation for efficient reverse transcription. Then, the sequencing libraries were established and an Agilent BioAnalyzer 2100 instrument was employed to quantify the libraries. Next, the standard small RNA sequencing was performed through an Illumina NextSeq instrument, and the sequencing type was 50‐bp single‐read at 10 M reads. Finally, the Arraystar tRF & tiRNA‐seq data analysis package was used for further data analysis. The original raw data for the RNA‐seq analysis had been uploaded to the public GEO database (https://www.ncbi.nlm.nih.gov/geo/), and the ID number was ‘GSE181092’.

### 2.5 Immunofluorescence staining assay

The AR42J cell samples were fixed with 4% paraformaldehyde for 1h, and the above cells were subjected to 0.1% Triton X‐100 for 30 min to improve permeability. Then, the antigens were blocked by treating cells with 10% goat serum and were incubated with the primary caspase‐1 antibody (Cell Signaling Technology, MA, USA) at 4℃ overnight. Next, the cells were incubated with the secondary antibody (Invitrogen, USA) for 1 h at room temperature without light. Finally, the cells were stained with 4′6‐diamidino‐2‐phenylindole (DAPI, 1:5000, Invitrogen, USA) to visualize the nucleus, and the fluorescence intensity was photographed and measured using a fluorescence microscope (Olympus, Tokyo, Japan).

### 2.6 Analysis of cytokines secretion by ELISA

The expressions of the inflammatory cytokines (IL‐6, IL‐13, TNF‐α, IL‐1β and IL‐18) were measured by using the corresponding ELISA kits purchased from R&D systems (MN, USA) in keeping with their instructions.

### 2.7 Examination of cell proliferation and viability

AR42J cells were cultured in the 96‐well plates with the density of 3000 cells per well, and each group had at least three repetitions. The cells were cultured in the incubator for differential time points and were incubated with 10 μl of MTT solution (Sigma, USA) for 4 h at 37℃, and the supernatants were removed. The cells were resolved with 150 μl of DMSO (Sigma, USA) and were fully vortexed. A microplate reader (Thermo Fisher Scientific, USA) was employed to measure the optical density (OD) values at the absorbance of 570 nm. In addition, the AR42J cells were stained with trypan blue staining dye, and the dead blue cells were counted under a light microscope to evaluate cell viability.

### 2.8 Dual‐luciferase reporter gene system assay

The binding sites in tRF3‐Thr‐AGT (3'‐CCT CCG TGG CGA CCC TA‐5’) and 3′ untranslated regions of ZBP1 mRNA (5'‐TTC CTG AGT GCT GGG AT‐3’) were predicted by performing bioinformatics analysis, and the binding sites in ZBP1 mRNA were mutated as 5′‐TTC CTG CAG CAA TCC CT‐3′. The above wild‐type and mutant ZBP1 sequences were cloned into the luciferase vectors, which were further co‐transfected with tRF3‐Thr‐AGT mimic into the AR42J cells by using the Vector Transfection reagent (QIAGEN, CA, USA) following the manufacturer's protocol. At 48 h post‐transfection, luciferase activities were measured by using the dual‐luciferase reporter gene system (Promega, WI, USA).

### 2.9 RNA pull‐down assay

A biotin‐labelled ZBP1 probe (5′‐TGA AGT CCC ACA TTC CTG AGT GCT GGG AT‐3′) was designed and synthesized by Sangon Biotech (Shanghai, China), which were used to validate the targeting relationship between ZBP1 and tRF3‐Thr‐AGT. Specifically, the AR42J cells were lysed and centrifuged, and the supernatants were collected and were used as input. The lysates were next incubated with the biotin‐labelled ZBP1 probe‐streptavidin Dynabeads (Invitrogen, USA) at 30℃ overnight. Then, the above mixtures were washed and the formaldehyde‐crosslinking was reversed by co‐treating cells with Proteinase K, and the following Real‐Time qPCR was performed to examine the pull‐down efficiency for tRF3‐Thr‐AGT.

### 2.10 Establishment of AP rat models

The male SD rats (*N* = 12) with 3–4 weeks of age were obtained from Research Animal Center Affiliated to Harbin Medical University, and the rats were fed under standard conditions with 12 h light/dark circle at 25℃. According to the experimental protocols provided by the previous work,^40^ the rats were anaesthetized with pentobarbital (3%) and SAP was induced by 5% sodium taurocholate (STC, 1 ml/kg, Sigma, USA) in a retrograde infusion manner into the biliopancreatic duct. The rats in the control group were treated with the same volume of saline solution. At 3 days post‐induction, the pancreas tissues and rats serum were collected for further analysis. The above animal experiments had been approved by the Ethics Committee Affiliated to Peking University People's Hospital (No. 201902332) and the Second Affiliated Hospital of Harbin Medical University (No. KY2021‐245).

### 2.11 Data analysis and visualization

Data analysis was conducted by using the SPSS 18.0 software and visualized by using the GraphPad Prism 8.0 software. Specifically, means from two groups were compared by using Student's *t* test, and one‐way ANOVA analysis was employed to analyse the statistical significance of the means from multiple groups (>2). The post hoc tests (Tukey test) was conducted after one‐way ANOVA analysis for correction test. *p* < 0.05 was regarded as statistical significance and marked by ‘*’.

## 3 RESULTS

### 3.1 The tsRNA, tRF3‐Thr‐AGT, was aberrantly downregulated in the cellular and animal AP models

**FIGURE 1:**
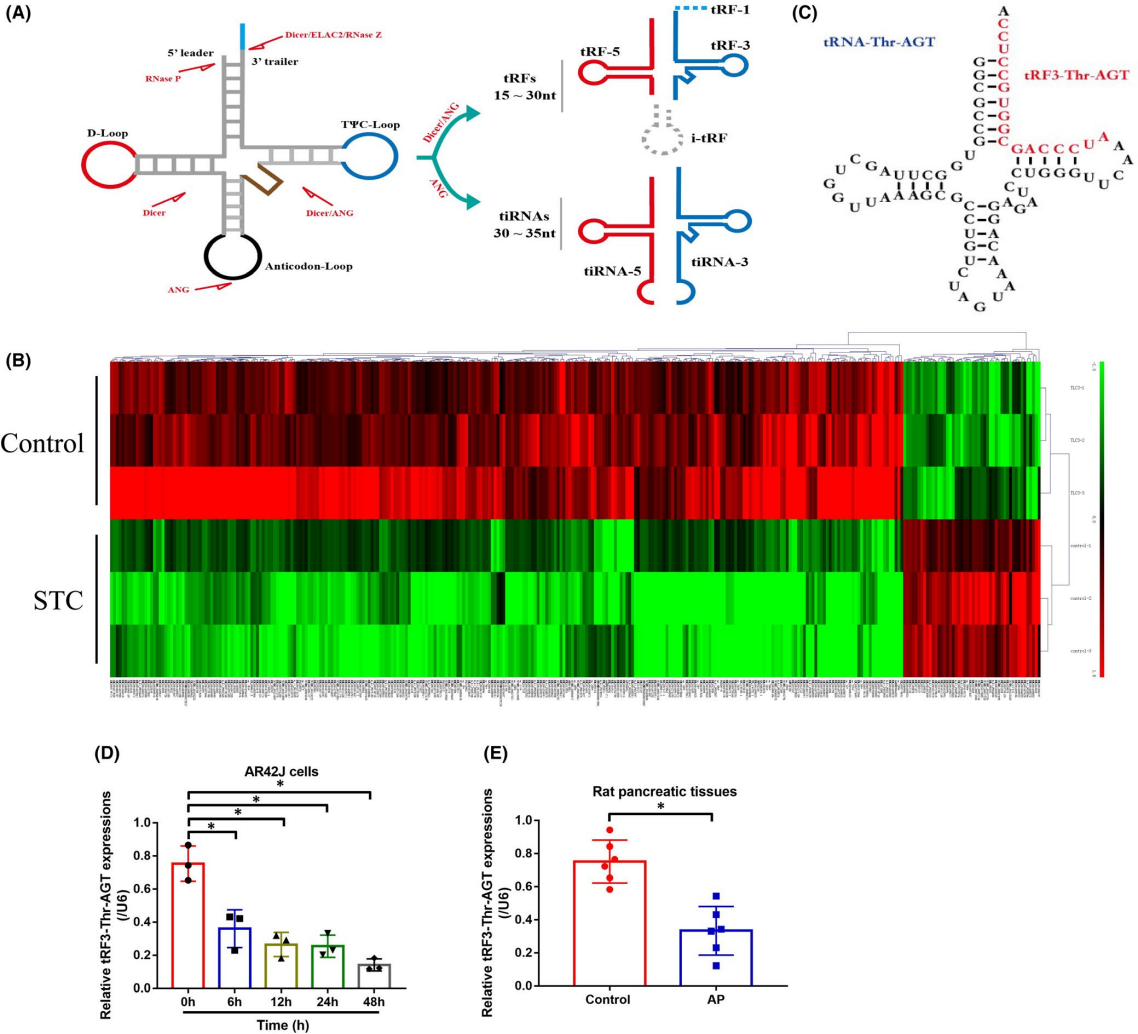
Association of tRF3‐Thr‐AGT levels with AP progression. (A) The generation process of tsRNAs. (B) The tsRNA sequencing results were shown in heat map. (C) The structure and sequence information for tRF3‐Thr‐AGT. Real‐Time qPCR was performed to examine the expression levels of tRF3‐Thr‐AGT in (D) AR42J cells (each experiment repeated for three times), (E) rat pancreatic tissues (each group had 6 rats, and individual experiment was repeated for three times). *p < 0.05

The tsRNAs are recently identified as novel regulators for various diseases,^24^, ^25^, ^26^ but it is still unclear whether tsRNAs are involved in regulating AP progression. The generation process of tsRNAs is shown in Figure 1A, and according to the experimental protocols provided by the previous literature,^40^ we established the cellular and animal models for AP by using the STC treatment method. Next, by performing the RNA‐seq analysis, we noticed that STC significantly altered the expression patterns of various tsRNAs in the AR42J cells. Especially, one novel tsRNA tRF3‐Thr‐AGT was significantly downregulated with biggest fold changes (Fold change = −167.04, *p* < 0.0005) after STC stimulation (Figure 1B), and the following bioinformatics analysis suggested that the potential downstream targets of tRF3‐Thr‐AGT were associated with AP progression (Figure S1). Thus, tRF3‐Thr‐AGT was selected for further analysis in the present study. The information regarding to location and sequence of tRF3‐Thr‐AGT are shown in Figure 1C, and the above results were validated by conducting the following Real‐Time qPCR analysis results that STC treatment (200 μM/L) decreased the expression levels of tRF3‐Thr‐AGT in AR42J cells in a time‐dependent manner (Figure 1D). In addition, the rat pancreatic tissues were collected, and our data supported that STC also downregulated tRF3‐Thr‐AGT in the tissues collected from AP rat but not the normal rat (Figure 1E).

### 3.2 STC treatment regulated cell viability, pyroptosis and inflammation in AP models

**FIGURE 2:**
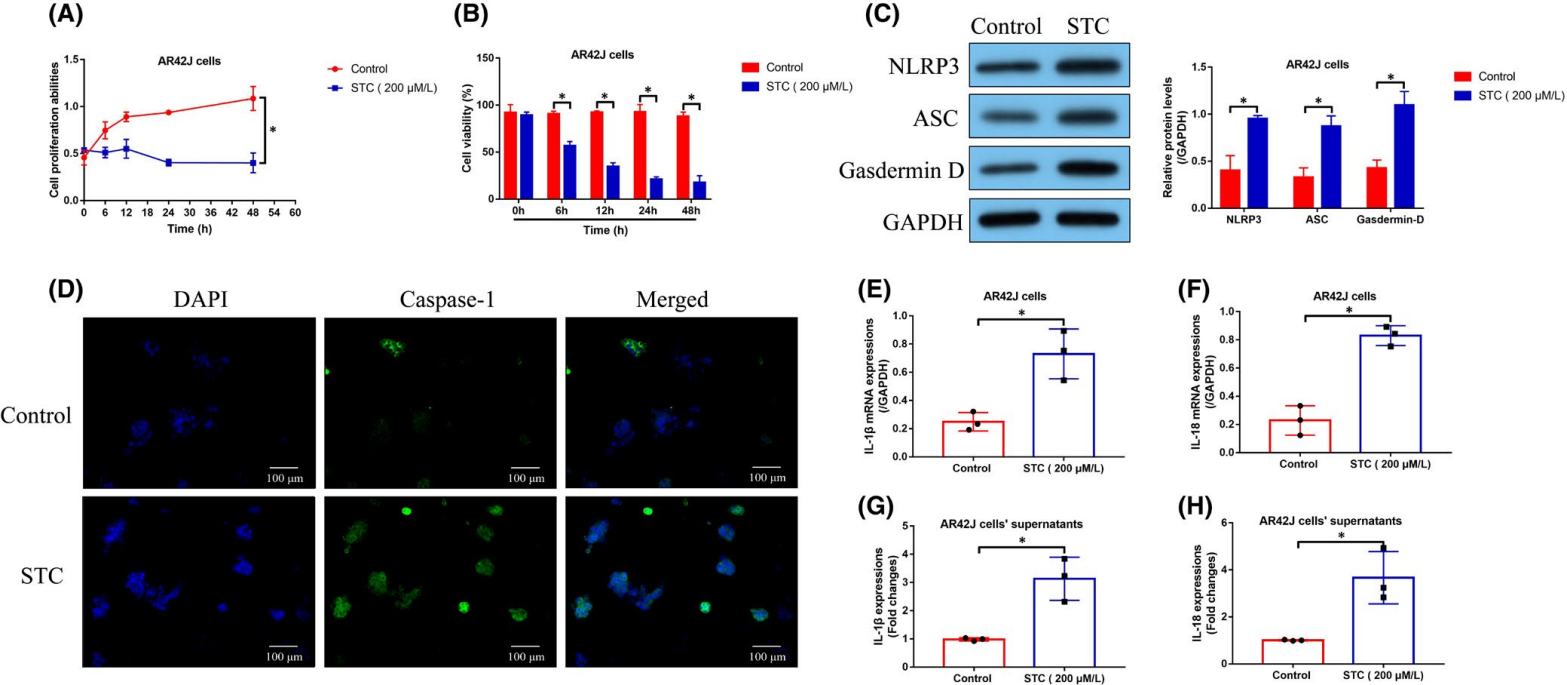
Cell pyroptosis was closely associated with AP development. (A) MTT assay and (B) trypan blue staining assay were, respectively, performed to examine cell proliferation and viability in AR42J cells (each experiment was repeated for three times). (C) The expression levels of NLRP3, ASC and Gasdermin D were determined by Western Blot analysis (each experiment was repeated for three times). (D) The expression levels and localization of caspase‐1 in AR42J cells were determined by immunofluorescence staining assay. (E, F) The mRNA and (G, H) protein levels of IL‐1β and IL‐18 were measured in AR42J cells and its supernatants (each experiment was repeated for three times). *p < 0.05

**FIGURE 3:**
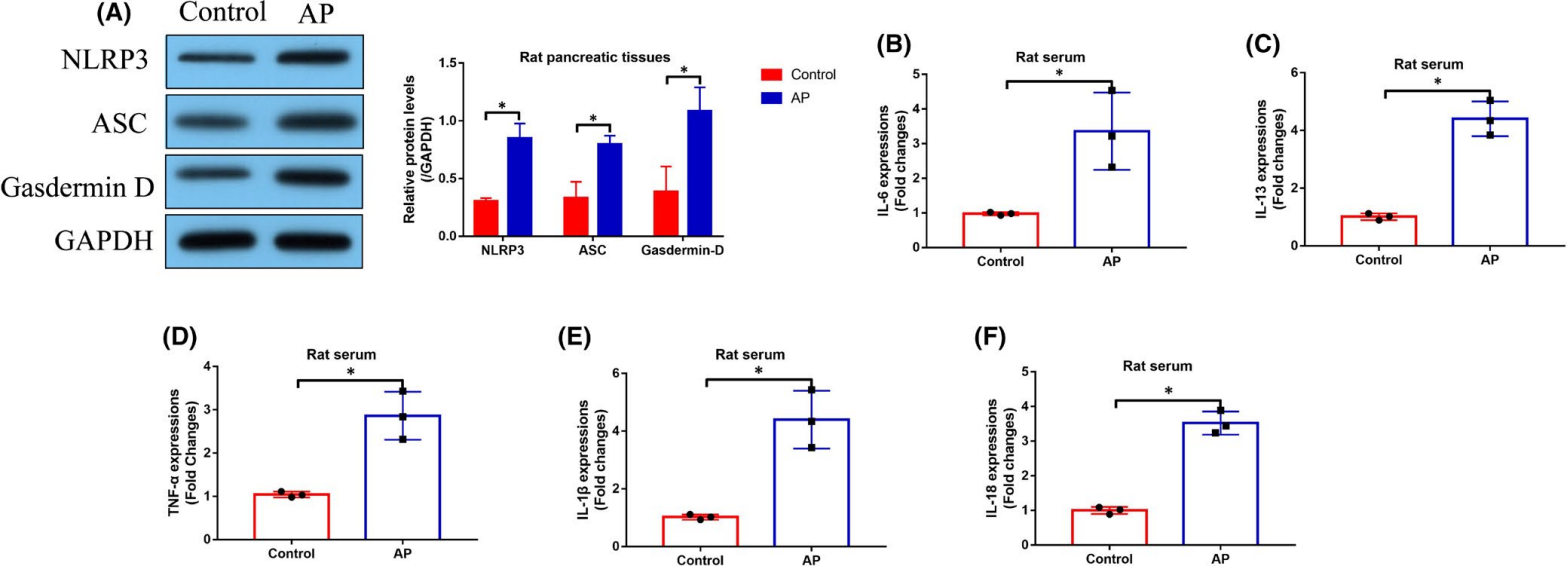
Cell pyroptosis and inflammation occurred during AP pathogenesis in vivo. (A) The rat pancreatic tissues were collected, and the expression levels of NLRP3, ASC, and Gasdermin D were examined by Western Blot analysis (each experiment was repeated for three times). The levels of the pro‐inflammatory cytokines in (B–F) rat serum (each group contained 3 rats). *p < 0.05

As previously reported,^14^, ^15^ cell pyroptosis and inflammation are closely associated with AP initiation and aggravation, but the detailed information in the existed database is still scarce. By performing the MTT assay (Figure 2A) and trypan blue staining assay (Figure 2B), we noticed that STC suppressed cell proliferation and viability in the AR42J cells in a time‐dependent manner. Also, the Western Blot analysis confirmed that STC upregulated NLRP3, ASC and Gasdermin D to promote pyroptotic cell death in the AR42J cells (Figure 2C), which were validated by the following immunofluorescence staining assay results that STC increased the expression levels of caspase‐1 in the cytoplasm of the AR42J cells (Figure 2D). Next, by performing the Real‐Time qPCR (Figure 2E, F) and ELISA (Figure 2G, H) analysis, we noticed that STC‐induced upregulation of IL‐1β and IL‐18 expressions in both AR42J cells and its supernatants. Consistently, the above cellular experiments were supported by the following animal results (Figure 3A‐F). Specifically, the expression levels of NLRP3, ASC and Gasdermin D in AP rat tissues (Figure 3A), and the pro‐inflammatory cytokines (IL‐6, IL‐13, TNF‐α, IL‐1β and IL‐18) in AP rat serum (Figure 3B‐F) were much higher than that of normal rats, indicating that cell pyroptosis and inflammation occurred during AP pathogenesis.

### 3.3 Overexpression of tRF3‐Thr‐AGT suppressed cell pyroptosis and inflammation in AR42J cells

**FIGURE 4:**
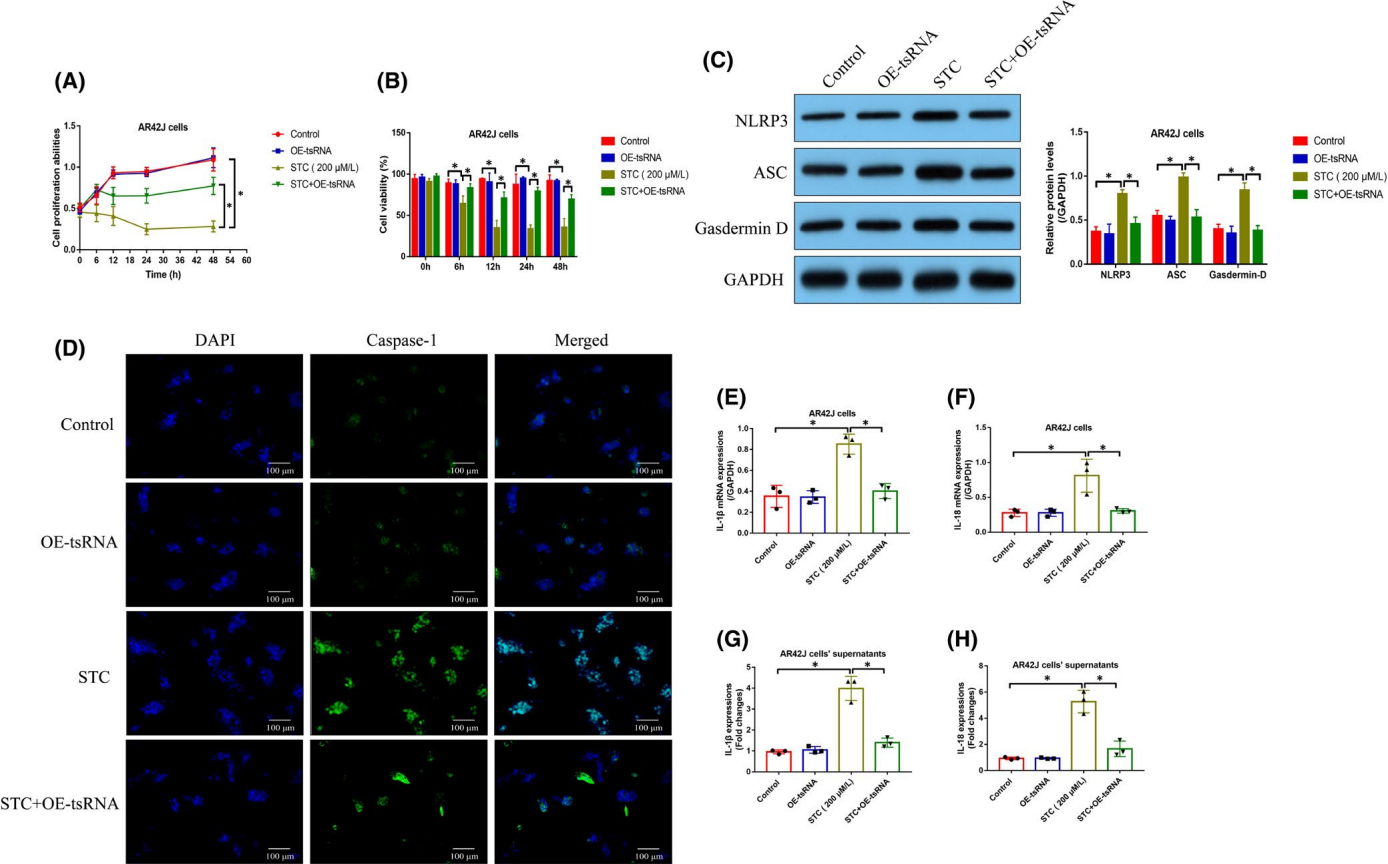
Upregulation of tRF3‐Thr‐AGT attenuated STC‐induced cell death and pyroptosis in AR42J cells. (A) MTT assay and (B) trypan blue staining assay were, respectively, used to determine cell proliferation and viability (each experiment was repeated for three times). The pyroptosis associated signatures were, respectively, examined by using (C) Western Blot analysis and (D) immunofluorescence staining assay. IL‐1β and IL‐18 expressions in (E, F) AR42J cells and (G, H) supernatants were measured by Real‐Time qPCR and ELISA. *p < 0.05

Since we had evidenced that tRF3‐Thr‐AGT was closely related with AP pathogenesis, and cell pyroptosis and inflammation are two pivotal factors that aggravate the development of AP, we next investigated whether tRF3‐Thr‐AGT directly regulated pyroptotic cell death and inflammation in AP models. To achieve this, the tRF3‐Thr‐AGT mimics were delivered into the AR42J cells (Figure S2A), which were subsequently treated with STC to induce cellular AP models. The cells were divided into groups as follows: Control, STC group, OE‐tRF3‐Thr‐AGT group, and STC plus OE‐tRF3‐Thr‐AGT group. As shown in Figure 4A, the MTT assay results showed that overexpression of tRF3‐Thr‐AGT alone did not influence cell proliferation in AR42J cells, but tRF3‐Thr‐AGT upregulation significantly rescued cell proliferation in AR42J cells treated with STC (Figure 4A). Also, the trypan blue staining assay results supported that STC‐induced inhibition of AR42J cell viability was also abrogated by upregulating tRF3‐Thr‐AGT (Figure 4B). Next, the Western Blot analysis was performed, and we found that STC upregulated NLRP3, ASC and Gasdermin D to promote cell pyroptosis in AR42J cells, which were reversed by upregulating tRF3‐Thr‐AGT (Figure 4C). The above results were also supported by the immunofluorescence staining assay results (Figure 4D), which showed that tRF3‐Thr‐AGT overexpression suppressed caspase‐1 expressions in AR42J cells. Next, the expression levels of the pro‐inflammatory cytokines (IL‐1β and IL‐18) were examined by conducting Real‐Time qPCR and ELISA analysis in AR42J cells and its supernatants, and our findings indicated that upregulation of tRF3‐Thr‐AGT attenuated STC‐induced cellular inflammation in AR42J cells (Figure 4E–H), implying that tRF3‐Thr‐AGT participated in the regulation of cell pyroptosis and inflammation during AP progression in vitro.

### 3.4 NLRP3‐deficiency reversed STC‐induced cell death and inflammation in AR42J cells

**FIGURE 5:**
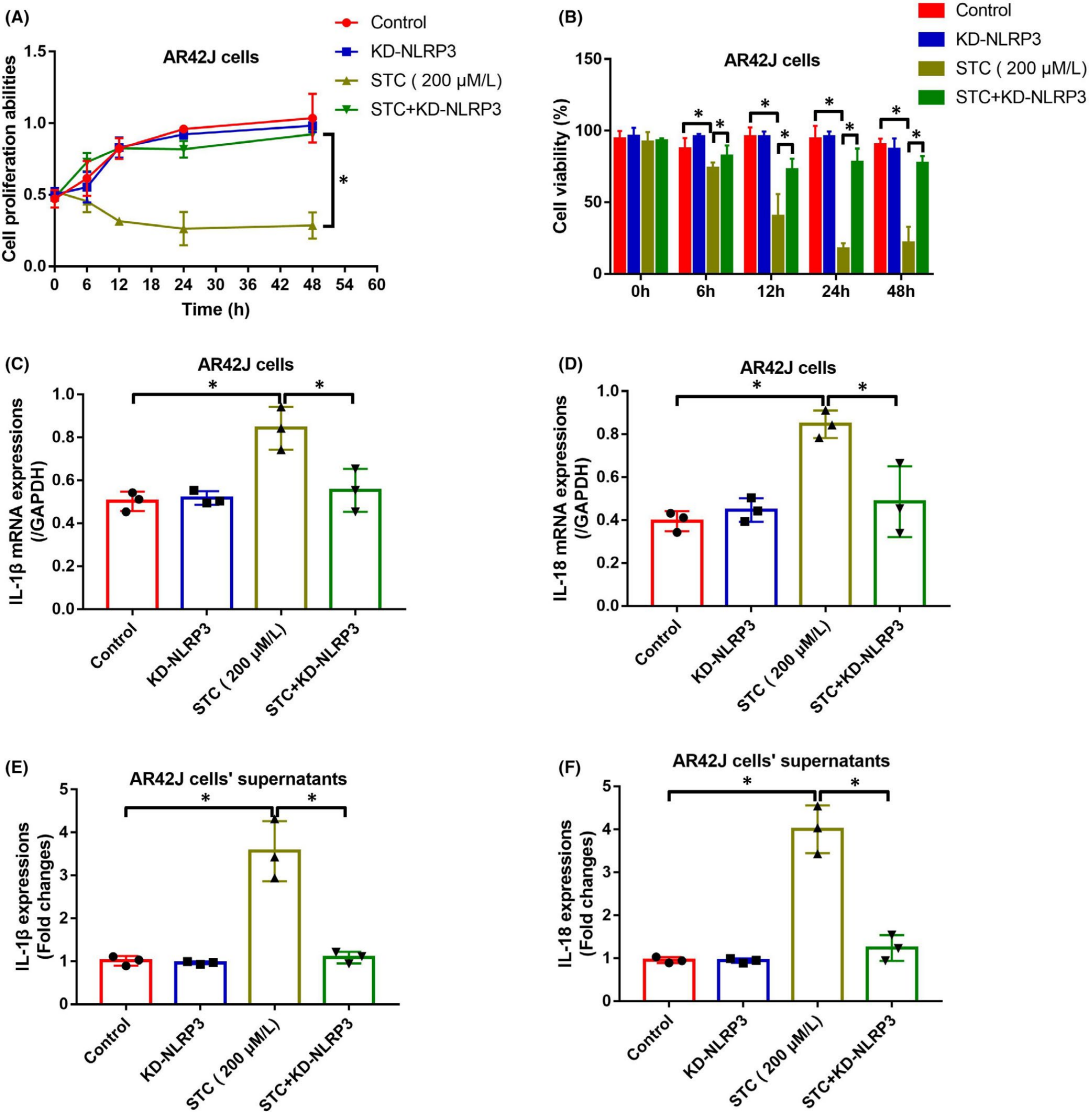
NLRP3‐deficiency reversed STC‐induced cell pyroptosis and inflammation in AR42J cells. Knock‐down of NLRP3 rescued cell (A) proliferation and (B) viability in STC‐treated AR42J cells (each experiment repeated for three times). (C, D) The generation and (E, F) secretion of IL‐1β and IL‐18 were suppressed by NLRP3‐deficiency in STC‐treated AR42J cells and its supernatants (each experiment repeated for three times). *p < 0.05

We next investigated whether targeting NLRP3‐mediated pyroptotic cell death was effective to restore cellular functions in the cellular AP models. To explore this issue, the NLRP3 knockdown vectors were initially delivered into the cells to establish NLRP3‐deficient AR42J cells (Figure S2B), which were subsequently exposed to STC for AP models induction. As shown in Figure 5A, B, downregulation of NLRP3 significantly rescued cell proliferation and viability in STC‐treated AR42J cells, as determined by MTT assay and trypan blue staining assay. In addition, knockdown of NLRP3 suppressed the mRNA and protein levels of IL‐1β and IL‐18 in both AR42J cells (Figure 5C, D) and its supernatants (Figure 5E, F), suggesting that targeting NLRP3‐mediated pyroptotic cell death was novel to attenuate STC‐induced AP progression in vitro.

### 3.5 TRF3‐Thr‐AGT inactivated NLRP3‐mediated pyroptotic cell death via suppressing ZBP1

**FIGURE 6:**
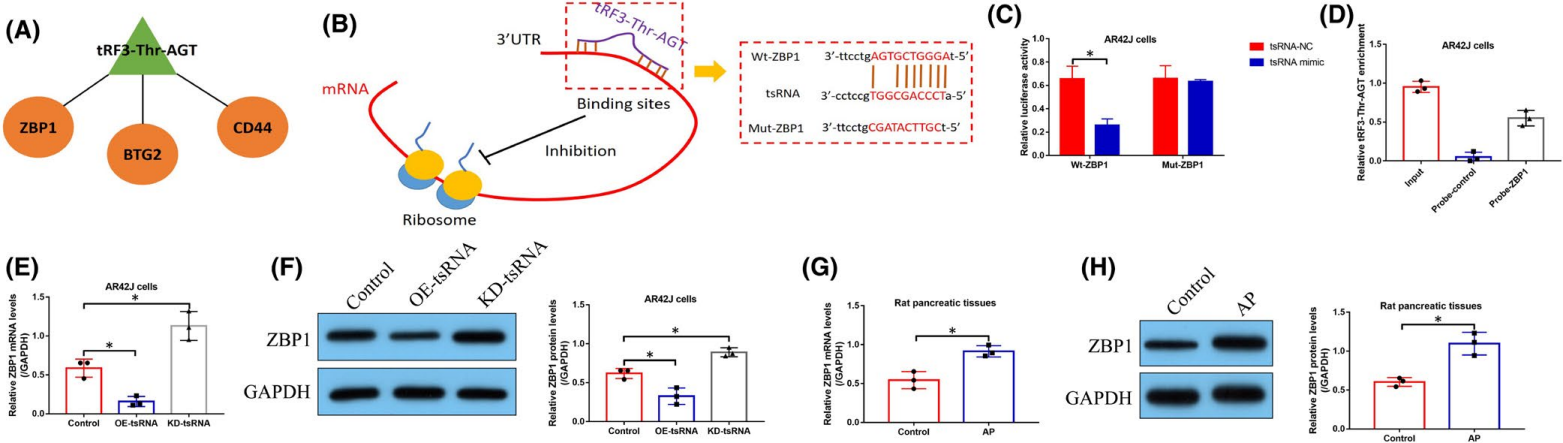
The regulatory mechanisms of tRF3‐Thr‐AGT and ZBP1. (A) The potential downstream targets for tRF3‐Thr‐AGT were predicted by using the bioinformatics analysis. (B) The binding sites between tRF3‐Thr‐AGT and 3′UTR of ZBP1 mRNA were predicted, which were validated by using the following (C) dual‐luciferase reporter gene system assay and (D) RNA pull‐down assay (each experiment repeated for three times). tRF3‐Thr‐AGT negatively regulated ZBP1 at both (E) mRNA and (F) protein levels (each experiment repeated for three times). (G, H) ZBP1 was upregulated in the AP rats pancreatic tissues, in contrast with the normal rats (each group had 3 rats, and individual experiment was repeated for three times). *p < 0.05

**FIGURE 7:**
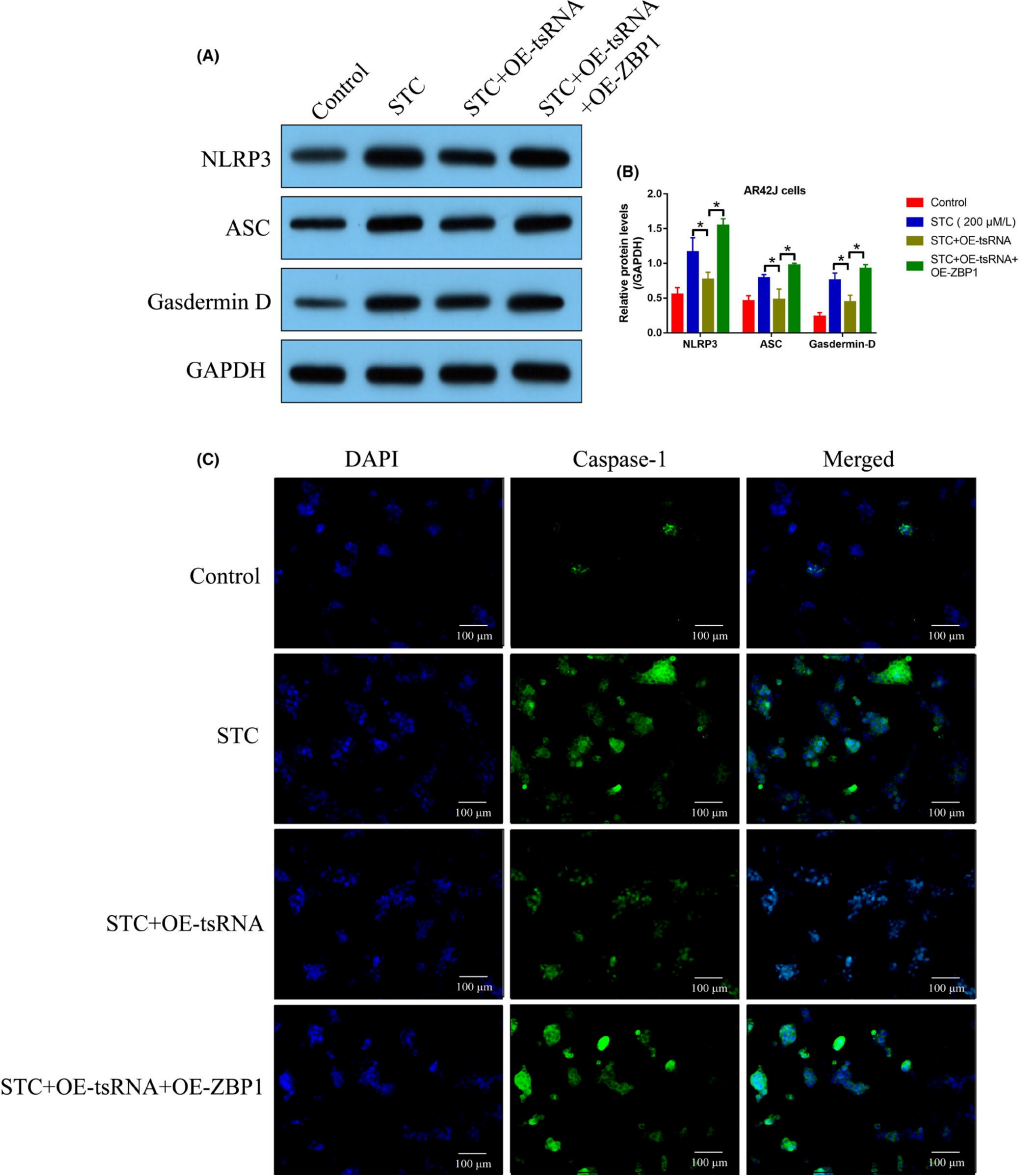
Overexpression of tRF3‐Thr‐AGT suppressed STC‐induced cell pyroptosis by downregulating ZBP1. (A, B) The expression levels of NLRP3, ASC and Gasdermin D were determined by Western Blot analysis (each experiment was repeated for three times). (C) Immunofluorescence staining assay was performed to determine the expression levels and localization of caspase‐1 in AR42J cells. *p < 0.05

Based on the fact that tRF3‐Thr‐AGT is capable of regulating NLRP3‐mediated pyroptotic cell death during AP pathogenesis, we next investigated the potential underlying mechanisms. By performing the bioinformatics analysis incorporated the data from miRanda and miRcode, we noticed that tRF3‐Thr‐AGT could bind to the 3′ untranslated regions (3′UTR) of CD44, BTG2 and ZBP1 (Figure 6A; Figure S1), which indicated the potential regulatory relationship. Interestingly, previous data suggest that ZBP1, instead of other proteins, is able to activate NLRP3‐mediated cell pyroptosis.^34^, ^35^ Thus, we selected ZBP1 for further investigations, and according to the principles of ceRNA network mechanisms, we hypothesized that tRF3‐Thr‐AGT might target 3′UTR of ZBP1 for its degradation, resulting in the inactivation of NLRP3 inflammasome, and this speculation was validated by the following experiments (Figure 6B‐H). Specifically, the binding sites between tRF3‐Thr‐AGT and ZBP1 were predicted (Figure 6B), which were validated by conducting the following dual‐luciferase reporter gene system assay (Figure 6C) and RNA pull‐down assay (Figure 6D). Specifically, data in Figure 6C suggested that the luciferase activities were significantly suppressed by tsRNA mimic in the AR42J cells co‐transfected with ZBP1 luciferase vectors, and the following experiments in Figure 6D indicated that tRF3‐Thr‐AGT was enriched by biotin‐labelled ZBP1 probes. Subsequent results supported that ZBP1 could be negatively regulated by tRF3‐Thr‐AGT at both mRNA (Figure 6E) and protein levels (Figure 6F), which were partially supported by the animal experiments that ZBP1 tended to be enriched in AP rat pancreatic tissues but not in the normal rat (Figure 6G, H). Moreover, we evidenced that the inhibiting effects of tRF3‐Thr‐AGT overexpression on STC‐induced cell pyroptosis were abrogated by upregulating ZBP1 (Figure 7A‐C), implying that tRF3‐Thr‐AGT regulated ZBP1 to influence cell pyroptosis during AP development.

### 3.6 TRF3‐Thr‐AGT exerted its protective effects in STC‐treated AR42J cells by inactivating the ZBP1/NLRP3 pathway

**FIGURE 8:**
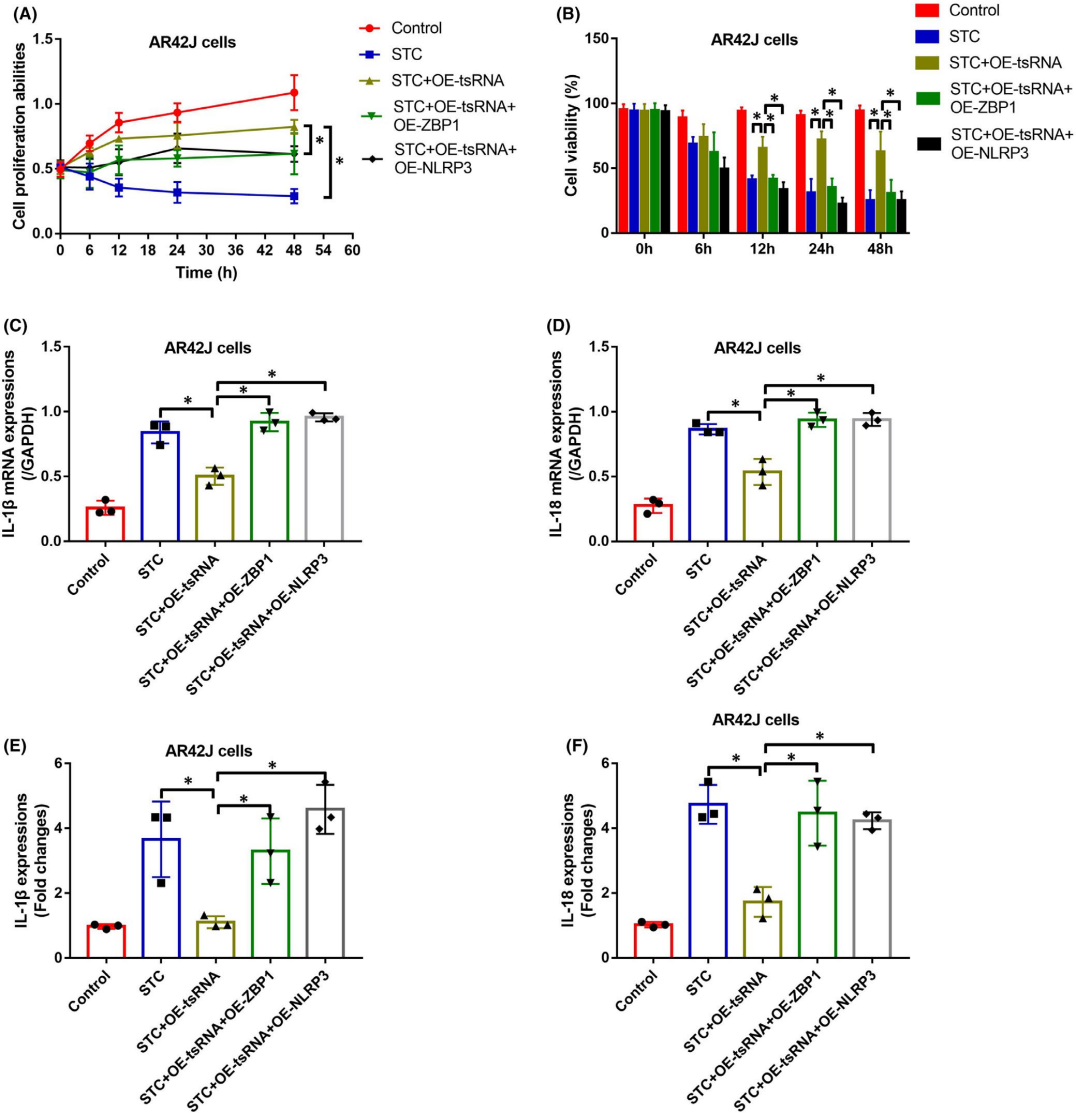
tRF3‐Thr‐AGT suppressed STC‐induced cell death and inflammation via modulating the ZBP1/NLRP3 pathway. (A, B) Cell proliferation and viability were measured by MTT assay and trypan blue staining assay (each experiment was repeated for three times). (C–F) The mRNA and protein levels of IL‐1β and IL‐18 in AT42J cells and its supernatants were measured by using Real‐Time qPCR and ELISA (each group had 6 rats, and individual experiment was repeated for three times). *p < 0.05

Finally, we investigated whether tRF3‐Thr‐AGT influenced the cellular functions in AP models through modulating the ZBP1/NLRP3 pathway. The AR42J cells were, respectively, pre‐transfected with tRF3‐Thr‐AGT mimic, and overexpression vectors for ZBP1 and NLRP3 (Figure S2A‐E), and Real‐Time qPCR and Western Blot analysis validated that the above vectors were successfully delivered into the cells. Then, the AR42J cells were exposed to STC to induce cellular models for AP, and the cells were grouped as follows: Control, STC group, STC + OE‐tRF3‐Thr‐AGT group, STC + OE‐tRF3‐Thr‐AGT + OE‐ZBP1 group, and STC + OE‐tRF3‐Thr‐AGT + OE‐NLRP3 group. As shown in Figure 8A, B, the MTT assay and trypan blue staining assay results suggested that overexpression of tRF3‐Thr‐AGT rescued cell proliferation and viability in STC‐treated AR42J cells, which were abrogated by upregulating both ZBP1 and NLRP3. In addition, we validated that tRF3‐Thr‐AGT upregulation suppressed the mRNA and protein levels of inflammation association cytokines, including IL‐1β and IL‐18, in AR42J cells and its supernatants by downregulating ZBP1 and NLRP3 (Figure 8C–F). The above data hinted that tRF3‐Thr‐AGT suppressed STC‐induced cell death and inflammation via inactivating the ZBP1/NLRP3 pathway.

## 4 DISCUSSION

Acute pancreatitis (AP) is a pancreas‐associated disease, which is featured with super‐inflammatory reactions, and 20% of AP patients are diagnosed as severe AP (SAP) with high mortality.^1^, ^2^, ^3^ Unfortunately, as the results of its complicated pathogenesis mechanisms, there is still no effective treatment strategies for SAP in clinic. Thus, this disease brings huge health burden for human beings.^4^, ^5^, ^6^, ^7^ As previously reported, NLRP3‐mediated pyroptotic cell death and inflammation are important factors that contribute to AP pathogenesis, and targeting this biological process is proved as an effective strategy to treat SAP.^14^, ^41^, ^42^ For example, data from Wu et al.,^41^ Wang et al.^14^ and Gao et al.^42^ evidence that activation of NLRP3‐mediated pyroptotic cell death facilitates AP aggravation, and Han et al. report that inhibition of myeloid‐specific dopamine D (2) receptor‐mediated inflammation is effective to ameliorate AP progression.^43^ The above research findings are supported by our study, which verified that cell pyroptosis and inflammation occurred during AP pathogenesis. Also, blockage of NLRP3‐mediated cell pyroptosis suppressed inflammation and ameliorated AP *in vitro*, indicating that inhibition of cell pyroptosis mediated inflammation was capable of ameliorating AP.

Recently, the non‐coding RNAs (ncRNAs) with post‐transcriptional activities, such as LncRNAs, circRNAs and miRNAs, are identified to be closely associated with various diseases, including AP.^44^, ^45^ Aside from the above ncRNAs, emerging shreds of evidence indicate that a newly identified endogenous transfer RNA (tRNA)‐derived small RNAs (tsRNAs) participate in the regulation of differential diseases and cellular functions. For example, Zhang et al. evidence that tsRNAs can be used as potential therapeutic biomarkers for Alzheimer's disease,^24^ Luo et al. explore the relationship between aberrant tsRNAs expressions and IgA nephropathy development,^25^ and other researchers report that tsRNAs participate in the regulation of cancer progression.^26^, ^46^, ^47^, ^48^ However, no literature report the involvement of tsRNAs in regulating AP pathogenesis. In this study, we firstly screened out a novel tsRNA tRF3‐Thr‐AGT that was aberrantly downregulated in AP, and upregulation of tRF3‐Thr‐AGT rescued cell viability, suppressed cell inflammation and pyroptosis to attenuate AP, which were partially supported by the previous work,^49^ indicating that tRF3‐Thr‐AGT was a potential diagnostic and therapeutic agent for AP treatment in clinic.

Next, we investigated the underlying mechanisms by which tRF3‐Thr‐AGT affects cell pyroptosis during AP progression. According to the existing information that tsRNAs share similar properties with miRNAs,^50^ we hypothesized that tsRNAs might also target the 3′ untranslated regions (3′UTRs) of their downstream target genes. Thus, by performing the bioinformatics analysis, we predicted that the 3′UTR of Z‐DNA‐binding protein 1 (ZBP1) could be targeted by tRF3‐Thr‐AGT, which was validated by the following experiments, suggesting that tRF3‐Thr‐AGT degraded ZBP1 by targeting its 3′ UTR. As previously described, ZBP1 is an innate sensor of viral infections, which is involved in the regulation of inflammatory cell death and inflammasomes activation.^51^ Of note, ZBP1 is capable of triggering pyroptotic cell death via activating NLRP3 inflammasome,^52^ which further aggravate the seriousness of influenza infection.^53^, ^54^ In this study, the suppressing effects of tRF3‐Thr‐AGT overexpression on cell pyroptosis and inflammation in AP models were abrogated by upregulating ZBP1, suggesting that tRF3‐Thr‐AGT exerted its protective effects during AP development by inhibiting ZBP1‐mediated cell pyroptosis.

## 5 CONCLUSIONS

Based on the above data, we have drawn the conclusions that upregulation of tRF3‐Thr‐AGT inactivated NLRP3‐mediated cell pyroptosis and inflammation by targeting the 3′UTR of ZBP1 for its degradation, resulting in the suppression of AP progression. This study firstly investigated the role and underlying mechanisms of a novel tRF3‐Thr‐AGT in regulating AP development, which broadened our knowledge in this field and provided possible diagnostic and therapeutic biomarkers for AP in clinic.

## CONFLICT OF INTEREST

Not applicable.

## AUTHOR CONTRIBUTIONS

**Boshi Sun:** Conceptualization (equal); Formal analysis (equal); Investigation (equal); Methodology (equal); Software (equal); Writing‐original draft (equal). **Zhuomiaoyu Chen:** Data curation (equal); Formal analysis (equal); Resources (equal); Software (equal); Visualization (equal). **Qiang Chi:** Formal analysis (equal); Methodology (equal); Resources (equal); Software (equal); Validation (equal). **Yifan Zhang:** Data curation (equal); Methodology (equal); Resources (equal); Software (equal); Validation (equal). **Bo Gao:** Conceptualization (equal); Funding acquisition (lead); Project administration (equal); Supervision (equal); Validation (equal); Writing‐review & editing (equal).

## ETHICS APPROVAL

The Ethics CommitteeAffiliated to Peking University People's Hospital and the Second AffiliatedHospital of Harbin Medical University approved our animal experiments.

## CODE AVAILABILITY

Not applicable.

## CONSENT TO PARTICIPATE

Not applicable.

## CONSENT FOR PUBLICATION

Not applicable.

## Supporting information

Figure S1

Figure S2

## Data Availability

All the data had been included in the manuscript.
