# Supplementary figures and images for: Endogenous tRNA‐derived small RNA (tRF3‐Thr‐AGT) inhibits ZBP1/NLRP3 pathway‐mediated cell pyroptosis to attenuate acute pancreatitis (AP)
Source: J Cell Mol Med. 2021 Oct 13;25(22):10441–53. doi: 10.1111/jcmm.16972 (PMC8581331; doi:10.1111/jcmm.16972)

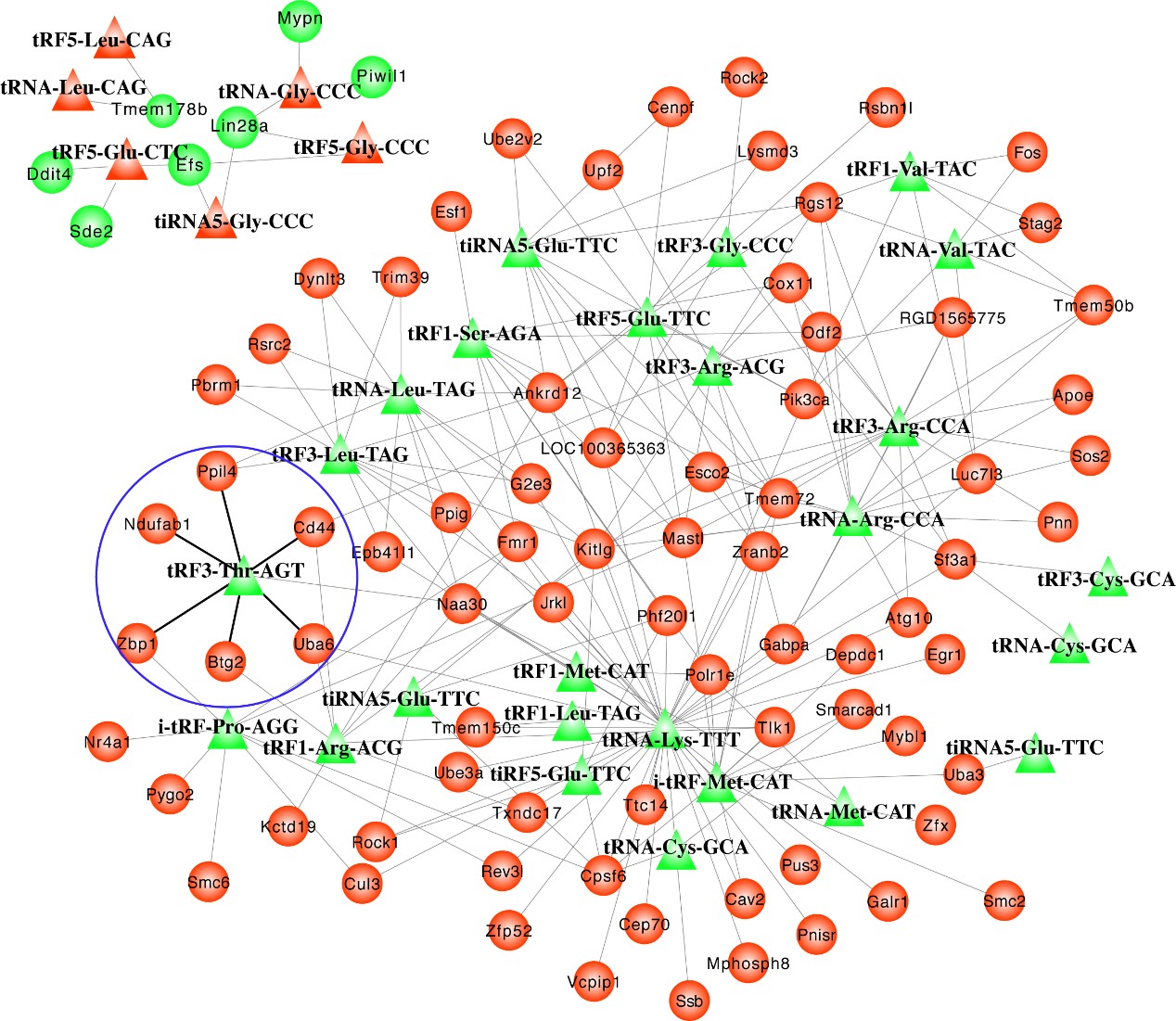

Supplement: Supplementary file 1 — Figure S1 [file JCMM-25-10441-s002.jpg]

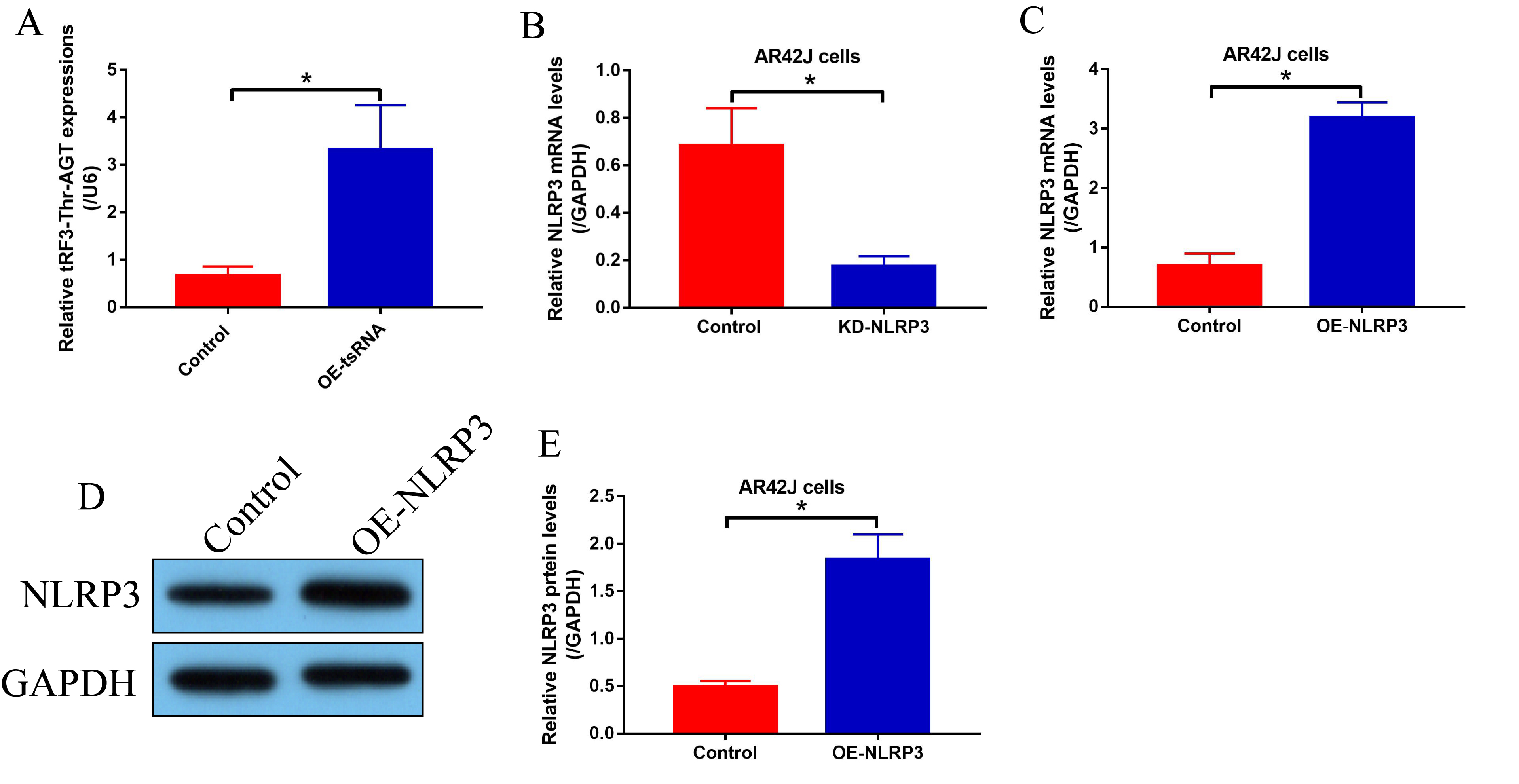

Supplement: Supplementary file 2 — Figure S2 [file JCMM-25-10441-s001.jpg]
